# A Low-Frequency Multi-Band Piezoelectric MEMS Acoustic Sensor Inspired by *Ormia ochracea*

**DOI:** 10.3390/mi16040451

**Published:** 2025-04-10

**Authors:** Yi Liu, Liye Zhao, Xukai Ding

**Affiliations:** School of Instrument Science and Engineering, Southeast University, Nanjing 210096, China; yiliu208@seu.edu.cn (Y.L.); ding.xk@seu.edu.cn (X.D.)

**Keywords:** *Ormia ochracea*, multi-band, low frequency, acoustic sensor, piezoelectric

## Abstract

Since the discovery of the unique auditory system of the *Ormia ochracea* fly, researchers have designed various directional acoustic sensors inspired by its principles. However, most of these sensors operate only within a single- or dual-frequency band and typically exhibit high eigenfrequencies, making them unsuitable for low-frequency applications. This paper proposes a low-frequency, multi-band piezoelectric MEMS acoustic sensor that incorporates an improved coupling structure within the inner diaphragm to enable low-frequency signal detection in a compact design. Additionally, an asymmetric wing and coupled structure are introduced in both the inner and outer diaphragms to achieve multi-band frequency response. Aluminum nitride (AlN), a material with low dielectric and acoustic losses, is selected as the piezoelectric material. The sensor operates in the d₃₃ mode and employs a branched comb-like interdigitated electrode design to enhance the signal-to-noise ratio (SNR). Simulation results demonstrate that the four eigenfrequencies of the sensor are evenly distributed below 2000 Hz, and at all eigenfrequencies, the sensor exhibits a consistent cosine response to variations in the incident elevation angle of the sound source.

## 1. Introduction

In daily production and everyday life, various acoustic signals carry rich information. As an essential research area in signal processing and sensing technology, sound source localization has found widespread applications in hearing aids, mobile robotics, and surveillance systems [1,2]. Traditional sound source localization methods primarily rely on omnidirectional microphone arrays arranged at uniform intervals. By measuring the time difference of arrival (TDOA) of sound signals at different microphones, the sound source position can be determined. However, this approach faces size constraints in miniaturized applications [3]. Since localization accuracy depends on the spacing between microphones, reducing the spacing improves the resolution of time delay measurements. However, when the spacing becomes smaller than the wavelength of the detected acoustic signal, diffraction and reflection effects significantly increase the complexity of signal acquisition and processing, ultimately affecting localization accuracy.

In recent years, researchers have drawn inspiration from the auditory systems of small biological organisms to overcome the limitations of conventional sound source localization techniques. In 1995, Miles et al. [4] reported the unique auditory system of *Ormia ochracea*, a species of fly capable of achieving high-precision sound localization (±2°) at a frequency of 5 kHz, despite having an interaural distance of only ~500 μm. This capability arises from its specialized inter-tympanal bridge structure, which couples the vibrations of both tympana, thereby enhancing interaural level difference (ILD) and interaural time difference (ITD) [5]. Due to the presence of the inter-tympanal bridge, the auditory system exhibits two primary vibration modes: the rocking mode, in which the two tympana oscillate out of phase, and the bending mode, in which they oscillate in phase.

Inspired by the auditory system of *Ormia ochracea*, researchers have developed various biomimetic acoustic sensors, which can be categorized into three main types: optical sensing [6,7], capacitive sensing [8,9,10], and piezoelectric sensing [11,12,13,14]. Among these, optical sensing offers high sensitivity but suffers from a complex optical system and large size, making miniaturization and commercialization challenging. Capacitive sensing, while structurally simple, requires a bias voltage and is prone to high residual noise due to comb finger misalignment caused by residual stress during fabrication [15]. In contrast, piezoelectric sensing does not require a bias voltage, features a more compact structure, and is cost-effective, making it an increasingly popular choice in the field of MEMS acoustic sensors.

Currently, most piezoelectric MEMS directional acoustic sensors use lead zirconate titanate (PZT) as the piezoelectric material. However, PZT exhibits high dielectric and acoustic losses, leading to significant thermo-mechanical noise. In comparison, aluminum nitride (AlN) has lower dielectric and acoustic losses and is compatible with complementary metal-oxide-semiconductor (CMOS) processes [16], making it a more ideal piezoelectric material. Typically, piezoelectric transduction is achieved through either the d₃₁ or d₃₃ mode. In the d₃₁ mode, electrodes are placed on the top and bottom of the piezoelectric thin film, and the output signal depends on the film thickness. In the d₃₃ mode, interdigitated electrodes (IDTs) are patterned on the surface of the piezoelectric thin film, and the output signal is determined by the electrode spacing, allowing for enhanced sensor sensitivity through optimized design [17].

Despite significant advancements in biomimetic acoustic sensor research in recent years, most existing designs operate only within a single- or dual-frequency band and exhibit weak responses at non-resonant frequencies. To date, only a few studies have focused on broadening the operational bandwidth [18,19]. Additionally, due to the inherent trade-off between sensor size and eigenfrequency, most designs have relatively high eigenfrequencies to achieve miniaturization, making them unsuitable for low-frequency acoustic applications.

To address these challenges, this paper proposes a low-frequency, multi-band piezoelectric MEMS acoustic sensor. The sensor employs an improved coupling structure to enable low-frequency signal detection in a compact form factor. By integrating an asymmetric wing design with a coupled diaphragm configuration, the sensor achieves multi-band operation. Furthermore, AlN, a piezoelectric material with low dielectric and acoustic losses, is selected and combined with the d₃₃ mode. The interdigitated electrode structure is optimized using a branched comb design to enhance the SNR.

The main contributions of this work are as follows:

1. A mechanical analysis is conducted to reveal the coupled vibration characteristics of the *Ormia ochracea* auditory system, demonstrating that the rocking mode is driven by the interaural pressure difference, while the bending mode is driven by the total pressure.
2. An improved inter-tympanal bridge coupling structure is proposed to enable low-frequency detection in a miniaturized design.
3. An asymmetric wing design is introduced to enhance the rocking mode response, while a coupled structure of two diaphragm pairs enables multi-band operation.
4. AlN is adopted as the piezoelectric material in the d₃₃ mode, and a branched comb interdigitated electrode structure is designed to improve the SNR.
5. Simulation results confirm that the sensor’s four eigenfrequencies are evenly distributed below 2000 Hz and exhibit a consistent cosine directional response to the incident sound source at all eigenfrequencies.

The findings of this study provide new insights for the development of compact low-frequency sound source localization systems and promote the application of biomimetic MEMS acoustic sensors in robotic audition, speech enhancement, and environmental monitoring.

## 2. Mechanical Analysis of the *Ormia ochracea* Auditory System

### 2.1. Mechanical Model Development

**Figure 1:**
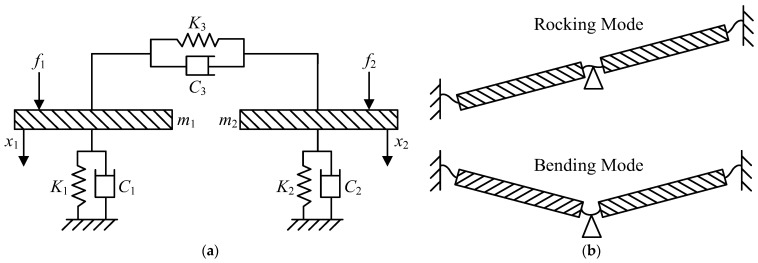
Mechanical model and vibration modes of the *Ormia ochracea* auditory system. (**a**) Two-degree-of-freedom mechanical model of the auditory system; (**b**) primary vibration modes of the auditory system.

In 1995, Miles et al. proposed a simplified mechanical model, demonstrating that the auditory system of *Ormia ochracea* can be fundamentally represented as a two-degree-of-freedom (2-DOF) system, as shown in Figure 1a. In this model, the masses of the two tympanic membranes are denoted as *m*_1_ and *m*_2_, their stiffness coefficients as *K*_1_ and *K*_2_, and their damping coefficients as *C*_1_ and *C*_2_, respectively. The inter-tympanal bridge, which serves as a coupling pivot, is approximated as a spring-mass system with stiffness coefficient *K*_3_ and damping coefficient *C*_3_.

When an external acoustic wave interacts with the auditory system, incident sound waves exert forces *f*_1_(*t*) and *f*_2_(*t*) on the two tympanic membranes, causing displacements *x*_1_(*t*) and *x*_2_(*t*), respectively. Based on the kinematic equations of a two-degree-of-freedom system, the dynamic equations of the system can be expressed as:

(1) $$\left[M\right][\ddot{x}(t)]+[C][\dot{x}(t)]+[K][x(t)]=[f(t)]$$

that is:

(2) $$\left[\begin{array}{cc} m_{1} & 0\\ 0 & m_{2} \end{array}\right]\left[\begin{array}{c} {\ddot{x}}_{1}(t)\\ {\ddot{x}}_{2}(t) \end{array}\right]+\left[\begin{array}{cc} C_{1}+C_{3} & C_{3}\\ C_{3} & C_{2}+C_{3} \end{array}\right]\left[\begin{array}{c} {\dot{x}}_{1}(t)\\ {\dot{x}}_{2}(t) \end{array}\right]+\left[\begin{array}{cc} K_{1}+K_{3} & K_{3}\\ K_{3} & K_{2}+K_{3} \end{array}\right]\left[\begin{array}{c} x_{1}(t)\\ x_{2}(t) \end{array}\right]=\left[\begin{array}{c} f_{1}(t)\\ f_{2}(t) \end{array}\right]$$

Since the mass of the tympanic membranes is significantly greater than that of the inter-tympanal bridge, it is reasonable to assume that the system’s kinetic energy is primarily concentrated in the tympanic membranes, allowing the mass of the inter-tympanal bridge to be neglected. Furthermore, to simplify the model, it is assumed that the two tympanic membranes have identical mass, stiffness coefficients, and damping coefficients, i.e., *m*_1_ = *m*_2_ = *m*, *K*_1_ = *K*_2_ = *K* and *C*_1_ = *C*_2_ = *C*. Under these assumptions, the frequency-domain displacement responses of the two tympanic membranes can be expressed as [20]:

(3) $$x_{1}\left(\omega \right)=\frac{f_{1}\left(\omega \right)\left[-\omega^{2}m+i\omega \left(C+C_{3}\right)+K+K_{3}\right]-f_{2}\left(\omega \right)\left(i\omega C_{3}+K_{3}\right)}{{\left[-\omega^{2}m+i\omega \left(C+C_{3}\right)+K+K_{3}\right]}^{2}-{\left(i\omega C_{3}+K_{3}\right)}^{2}}$$

(4) $$x_{2}(\omega )=\frac{f_{2}(\omega )\left[-\omega^{2}m+i\omega \left(C+C_{3}\right)+K+K_{3}\right]-f_{1}(\omega )\left(i\omega C_{3}+K_{3}\right)}{{\left[-\omega^{2}m+i\omega \left(C+C_{3}\right)+K+K_{3}\right]}^{2}-{\left(i\omega C_{3}+K_{3}\right)}^{2}}$$

Due to the coupling effect of the inter-tympanal bridge, the auditory system of *Ormia ochracea* exhibits two primary vibration modes, as illustrated in Figure 1b. These modes are: rocking mode—characterized by out-of-phase motion of the two tympanic membranes, corresponding to a lower eigenfrequency; bending mode—characterized by in-phase motion of the two tympanic membranes, corresponding to a higher eigenfrequency.

Under free vibration conditions, where the characteristic equation $\left|-\omega^{2}[M]+[K]\right|=0$ holds, the eigenfrequencies of the rocking mode *ω_r_* and the bending mode *ω_t_* can be expressed as:

(5) $$\omega_{r}=\sqrt{\frac{K}{m}},\omega_{t}=\sqrt{\frac{K+2K_{3}}{m}}$$

### 2.2. Relationship Between Tympanic Membrane Response and Sound Source Direction

The force exerted by an incident sound wave on the tympanic membrane surface can be expressed in exponential form as:

(6) $$f_{1}(\omega )=SPe^{i\omega \tau /2}{,f}_{2}(\omega )=SPe^{-i\omega \tau /2},$$

where *τ* represents the time delay of the sound wave reaching the two tympanic membranes, *P* is the incident sound pressure, and *ω* is the angular frequency. By solving the system using modal analysis [13], the frequency response of the tympanic membrane can be obtained as:

(7) $$\begin{array}{c} x_{1}\left(\omega \right) \end{array}=\frac{SP\cos\frac{\omega \tau }{2}}{m\left(\omega_{t}^{2}-\omega^{2}+2i\omega \xi_{t}\omega_{t}\right)}+\frac{\mathrm{iSPsin}\frac{\omega \tau }{2}}{m\left(\omega_{r}^{2}-\omega^{2}+2i\omega \xi_{r}\omega_{r}\right)}$$

(8) $$\begin{array}{c} x_{2}\left(\omega \right) \end{array}=\frac{SP\cos\frac{\omega \tau }{2}}{m\left(\omega_{t}^{2}-\omega^{2}+2i\omega \xi_{t}\omega_{t}\right)}-\frac{\mathrm{iSPsin}\frac{\omega \tau }{2}}{m\left(\omega_{r}^{2}-\omega^{2}+2i\omega \xi_{r}\omega_{r}\right)},$$

where $\xi_{r}=C/\left(2\omega_{r}m\right)$ and $\xi_{t}=\left(C+C_{3}\right)/\left(2\omega_{t}m\right)$ denote the damping ratios for the rocking mode and bending mode, respectively. When *ω* approaches the eigenfrequency of the rocking mode *ω_r_*, the displacement response is primarily driven by a term proportional to $\sin\frac{\omega \tau }{2}$. Conversely, when *ω* approaches the eigenfrequency of the bending mode *ω_t_*, the displacement response is primarily driven by a term proportional to $\cos\frac{\omega \tau }{2}$.

Let *θ* be the angle between the incident sound wave and the normal to the tympanic membrane, *d* be the distance between the centers of the two tympanic membranes, and *c* be the speed of sound. The time delay *τ* can be expressed as:

(9) $$\tau =\frac{dsin\theta }{c}$$

Since *τ* is small, the following approximations are held:

(10) $$sin\frac{\omega \tau }{2}=sin\left(\frac{\omega dsin\theta }{2c}\right)\approx \frac{\omega dsin\theta }{2c}$$

(11) $$cos\frac{\omega \tau }{2}=cos\left(\frac{\omega dsin\theta }{2c}\right)\approx 1-{\left(\frac{\omega dsin\theta }{2c}\right)}^{2}\approx 1$$

Thus, the displacement amplitudes of the tympanic membrane in the rocking and bending modes can be approximated as:

(12) $$A_{r}\approx \frac{SPsin\frac{\omega \tau }{2}}{m}\frac{1}{\sqrt{{\left(\omega_{r}^{2}-\omega^{2}\right)}^{2}+{\left(2\omega \xi_{r}\omega_{r}\right)}^{2}}}\propto sin\theta \propto \left|f_{1}(\omega )-f_{2}(\omega )\right|$$

(13) $$\begin{array}{l} A_{t}\approx \frac{SPcos\frac{\omega \tau }{2}}{m}\frac{1}{\sqrt{{\left(\omega_{t}^{2}-\omega^{2}\right)}^{2}+{\left(2\omega \xi_{t}\omega_{t}\right)}^{2}}}\approx constant\propto |f_{1}(\omega ){+f}_{2}(\omega )| \end{array}$$

This indicates that the rocking mode is primarily driven by the pressure difference between the two tympanic membranes, while the bending mode is driven by the sum of the pressures on both tympanic membranes. As a result, the displacement amplitude in the bending mode is significantly larger than that in the rocking mode, making the signal at the eigenfrequency of the rocking mode weaker and sometimes even difficult to detect.

## 3. Improved Coupling Structure Design

**Figure 2:**
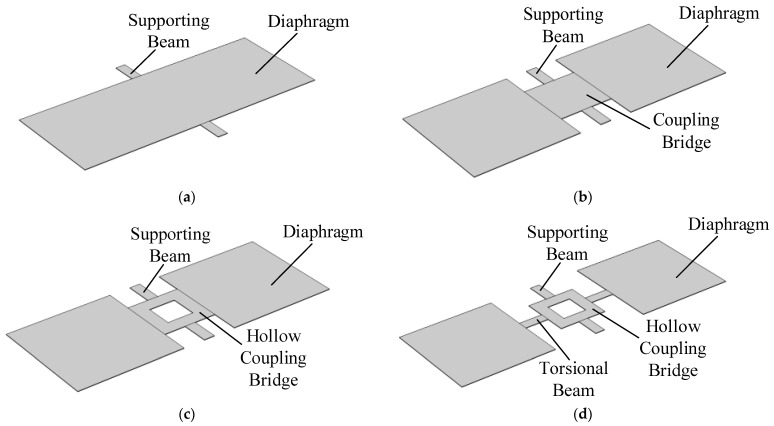
Structural schematic diagrams of different models. (**a**) Schematic diagram of Model A; (**b**) schematic diagram of Model B; (**c**) schematic diagram of Model C; (**d**) Schematic diagram of Model D.

Inspired by the inter-tympanal bridge structure in the auditory system of *Ormia ochracea*, conventional single-diaphragm directional acoustic sensors often adopt a seesaw-like structure. This configuration typically consists of a rectangular diaphragm supported by a pair of beams, which is defined in this study as Model A, as illustrated in Figure 2a. However, the eigenfrequency of such structures increases as the size decreases, posing challenges for detecting low-frequency signals while maintaining a compact design.

To address this issue, this study proposes an improved coupling structure that effectively reduces the eigenfrequency while keeping the diaphragm size comparable. This enhancement significantly improves the sensor’s ability to detect low-frequency signals. The following sections provide a detailed description of the design process of this coupling structure.

**Figure 3:**
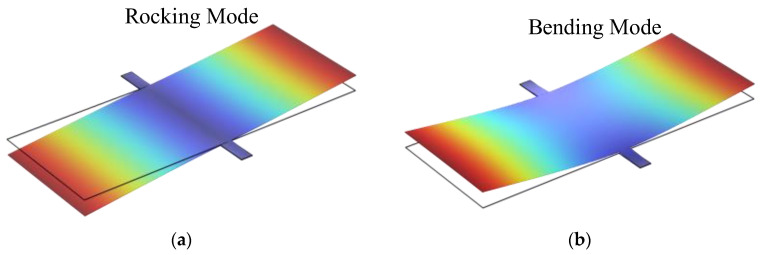
Vibration mode shapes of Model A under different modes. (**a**) Rocking mode; (**b**) bending mode.

First, the eigenfrequency analysis of Model A was performed using the finite element simulation software—COMSOL Multiphysics 6.1. The simulation results indicate that Model A exhibits the same vibration modes as the auditory system of *Ormia ochracea*, with its mode shapes shown in Figure 3. The calculated eigenfrequencies for the rocking and bending modes of Model A are 840.2 Hz and 1902.7 Hz, respectively.

Building upon Model A, the diaphragm was split into two separate sections and connected via a coupling bridge, forming Model B, as shown in Figure 2b. The dimensions of the coupling bridge were set to 1 mm × 1 mm, the two side flaps to 2 mm × 2 mm, and the supporting beams to 0.5 mm × 0.2 mm. Simulation results indicate that the eigenfrequencies of the rocking and bending modes in Model B were reduced to 832.1 Hz and 1600.9 Hz, respectively.

Building upon Model B, a hollowing treatment was applied to the coupling bridge, forming Model C, as shown in Figure 2c. The hollowed region was set to 0.5 mm × 0.5 mm, while other structural parameters remained unchanged. Simulation results show that the eigenfrequencies of the rocking and bending modes in Model C further decreased to 811.7 Hz and 1350.3 Hz, respectively.

**Figure 4:**
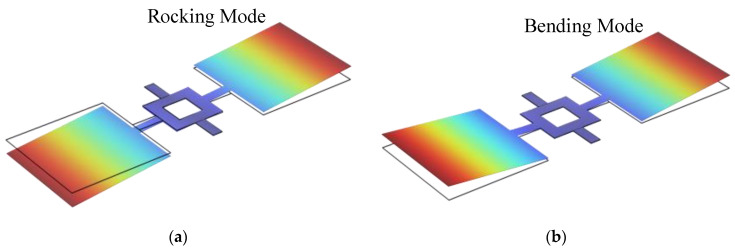
Vibration mode shapes of Model D under different modes. (**a**) Rocking mode; (**b**) bending mode.

Further modifications were made to Model C by adding a pair of torsional beams between the coupling bridge and the two side flaps, forming Model D, as illustrated in Figure 2d. The torsional beams were set to 0.5 mm × 0.2 mm, while all other structural parameters remained unchanged. Simulation results confirm that Model D retains the same vibration modes as the auditory system of *Ormia ochracea*, as shown in Figure 4. The calculated eigenfrequencies for the rocking and bending modes of Model D were further reduced to 490.8 Hz and 631.4 Hz, respectively.

To provide a clear comparison of the eigenfrequencies across different models, Table 1 presents the eigenfrequencies of the four models along with their respective reductions relative to Model A.

Comparison between Model B and Model A shows that by introducing a coupling bridge, both the rocking and bending mode eigenfrequencies of Model B decreased, with a more significant reduction observed in the bending mode. Comparison between Model C and Model B shows that by introducing perforations in the coupling bridge of Model B to form Model C, the eigenfrequencies of both modes were further reduced, again with a greater decrease in the bending mode. Comparison between Model D and Model C shows that by incorporating torsional beams into Model C to form Model D, the eigenfrequencies dropped substantially. Compared to the initial Model A, the rocking mode eigenfrequency decreased from 840.2 Hz to 490.8 Hz, a reduction of 41.6%, while the bending mode eigenfrequency decreased from 1902.7 Hz to 631.4 Hz, representing a 66.8% reduction.

In summary, the introduction of a perforated coupling bridge and torsional beams, i.e., the improved coupling structure, effectively reduces the eigenfrequencies of inter-tympanal bridge-based acoustic sensors, thereby enabling low-frequency signal detection in a compact design. This optimization facilitates sensor miniaturization and expands its applicability in low-frequency acoustic detection.

## 4. Multi-Band Response Design

### 4.1. Asymmetric Design for Enhanced Rocking Mode Response

According to the mechanical analysis of the *Ormia ochracea* auditory system in Section 2, the rocking mode is primarily driven by the pressure difference between the two sides, while the bending mode is driven by the sum of the pressures on both sides. Therefore, to enhance the sensor’s response in the rocking mode, the pressure difference can be amplified through an asymmetric design of the wing structures on both sides of the diaphragm. The following verification is performed using Model A and Model D as examples.

**Figure 5:**
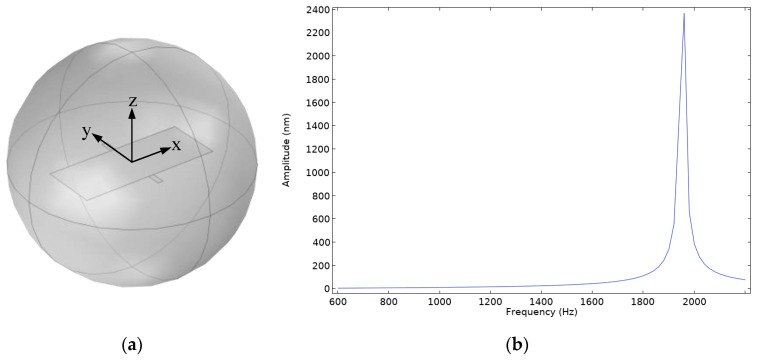
(**a**) Simulation model of the symmetric-wing Model A; (**b**) frequency response curve of the symmetric-wing Model A.

First, a simulation is conducted for Model A with symmetric wing structures, where the supporting beams are placed at the center of the diaphragm. Using COMSOL Multiphysics software, the diaphragm is positioned inside a damped spherical shell, and an incident sound source is applied outside the shell. Since the diaphragm’s size is much smaller than the distance between the sound source and the diaphragm in actual tests, the far-field condition is met. Therefore, the sound source in the simulation is set as a plane wave with a sound pressure amplitude of 1 Pa. The acoustic–structural coupling is modeled using a multi-physics approach that combines pressure acoustics and solid mechanics, with accurate physical field conditions, boundaries, and sound–structure interfaces defined. A frequency domain study is then established to calculate the amplitude variations of the diaphragm in the frequency range of 600–2200 Hz, and a frequency response curve is plotted. The simulation model and frequency response curve are shown in Figure 5.

**Figure 6:**
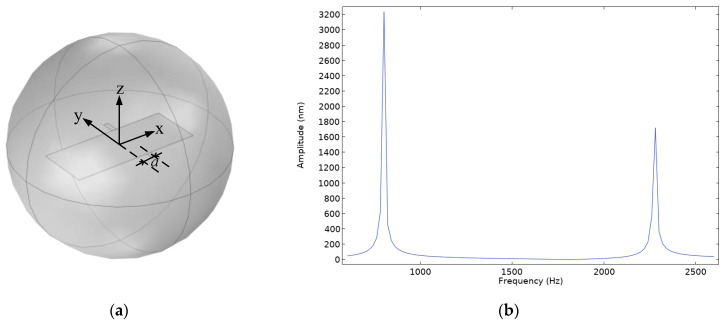
(**a**) Simulation model of the asymmetric-wing Model A; (**b**) Frequency response curve of the asymmetric-wing Model A.

From Figure 5, it can be observed that the displacement of the diaphragm in the rocking mode is significantly smaller than that in the bending mode. For Model A, increasing the area of one side’s wing and offsetting the supporting beam have an equivalent effect in amplifying the pressure difference. However, offsetting the supporting beam is more convenient. Therefore, the supporting beam of Model A is shifted in the positive x-axis direction, with an offset *a* of 0.5 mm, and a frequency domain study is conducted under the same simulation conditions to calculate the amplitude variations of the diaphragm in the frequency range of 600–2600 Hz, and a frequency response curve is plotted. The simulation model and frequency response curve are shown in Figure 6.

From Figure 6, it can be seen that, compared to the case where the supporting beam is not offset, the displacement of the diaphragm in the rocking mode increases significantly. Therefore, it can be concluded that, for Model A, appropriately offsetting the supporting beam can effectively enhance the sensor’s response in the rocking mode, thereby broadening its operating frequency range.

**Figure 7:**
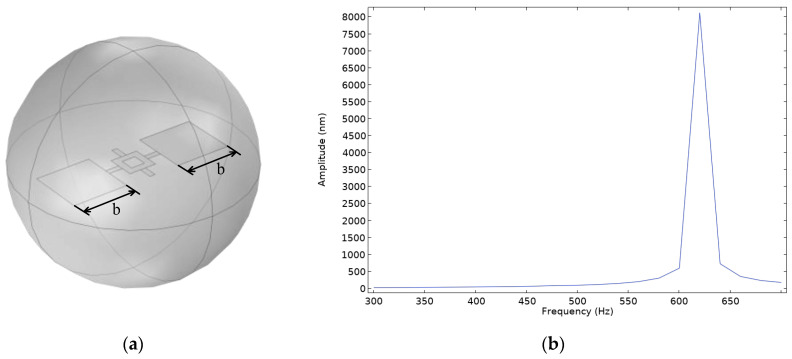
(**a**) Simulation model of the symmetric-wing Model D; (**b**) frequency response curve of the symmetric-wing Model D.

Next, a simulation is conducted for Model D with symmetric wing structures, where the areas of the wings on both sides of the diaphragm are equal, and the side lengths *b* of both wings are 2 mm. Using the same simulation conditions, a frequency domain study is performed to calculate the amplitude variations of the diaphragm in the frequency range of 300–700 Hz, and the frequency response curve is plotted. The simulation model and frequency response curve are shown in Figure 7.

**Figure 8:**
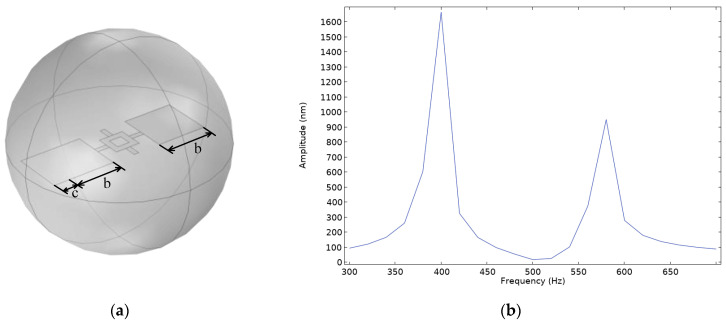
(**a**) Simulation model of the asymmetric-wing Model D; (**b**) frequency response curve of the asymmetric-wing Model D.

From Figure 7, it can be observed that the displacement of the diaphragm in the rocking mode remains significantly smaller than that in the bending mode. To enhance the response in the rocking mode, the length of the left wing in Model D is increased, with an increment *c* of 0.5 mm. A frequency domain study is then conducted under the same simulation conditions to compute the amplitude variations of the diaphragm within the frequency range of 300–700 Hz. The simulation model and frequency response curve are shown in Figure 8.

From Figure 8, it is evident that compared to the original configuration without an extended left wing, the diaphragm’s displacement in the rocking mode has increased significantly. Therefore, it can be concluded that for Model D, appropriately increasing the area of one wing effectively enhances the sensor’s response in the rocking mode, thereby expanding its operational frequency range.

### 4.2. Coupled Design of Inner and Outer Diaphragms

To broaden the operational frequency range of the sensor, a coupled diaphragm configuration can be implemented by integrating two sets of diaphragms. By combining Model A and Model D, four different coupled structures can be designed as follows:

1. Coupled structure A where both the inner and outer diaphragms adopt Model A;
2. Coupled structure B where both the inner and outer diaphragms adopt Model D;
3. Coupled structure C where the inner diaphragm adopts Model A, while the outer diaphragm adopts Model D;
4. Coupled structure D where the inner diaphragm adopts Model D, while the outer diaphragm adopts Model A.

**Figure 9:**
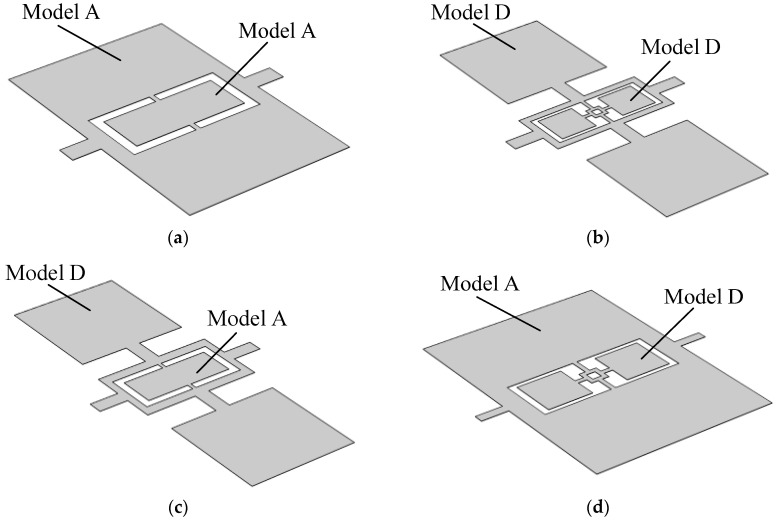
(**a**) Schematic diagram of the coupled structure A; (**b**) schematic diagram of the coupled structure B; (**c**) schematic diagram of the coupled structure C; (**d**) schematic diagram of the coupled structure D.

The schematic diagrams of these four coupled structures are illustrated in Figure 9.

To analyze the dynamic characteristics of these structures, eigenfrequency simulations were conducted for each configuration, yielding the following results:

- Coupled structure A where the eigenfrequencies of the outer diaphragm in the rocking and bending modes are 636.0 Hz and 956.7 Hz, respectively, while those of the inner diaphragm are 2894.4 Hz and 8458.2 Hz, respectively;
- Coupled structure B where the eigenfrequencies of the outer diaphragm in the rocking and bending modes are 186.5 Hz and 232.3 Hz, respectively, while those of the inner diaphragm are 1719.7 Hz and 2664.6 Hz, respectively;
- Coupled structure C where the eigenfrequencies of the outer diaphragm in the rocking and bending modes are 189.6 Hz and 246.9 Hz, respectively, while those of the inner diaphragm are 2958.9 Hz and 8763.4 Hz, respectively;
- Coupled structure D where the eigenfrequencies of the outer diaphragm in the rocking and bending modes are 451.6 Hz and 870.8 Hz, respectively, while those of the inner diaphragm are 1728.5 Hz and 2016.0 Hz, respectively.

A comparative analysis of the eigenfrequencies of the inner and outer diaphragms in the rocking and bending modes for the four coupled structures is summarized in Table 2.

**Figure 10:**
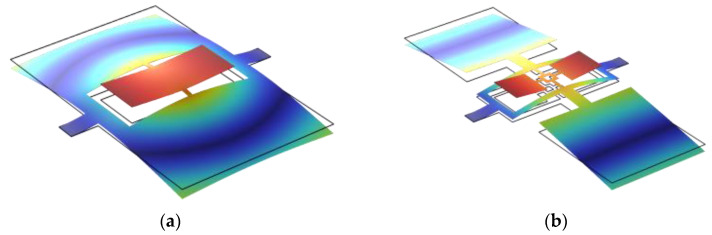
(**a**) Interference mode diagram of coupled structure A; (**b**) interference mode diagram of coupled structure B.

From Table 2, it can be observed that coupled structure D offers several advantages as follows:

1. Even distribution of eigenfrequencies where the four eigenfrequencies are evenly distributed across the low- and mid-frequency ranges;
2. Suitability for low-frequency detection where all eigenfrequencies are below 2000 Hz, making it well-suited for low-frequency signal detection;
3. Minimization of interfering modes where compared to other coupled configurations, coupled structure D exhibits purer eigenmodes within the target detection frequency range, reducing the likelihood of interference. For example, coupled structures A and B contain additional interfering modes, as illustrated in Figure 10, with eigenfrequencies of 2939.2 Hz and 1210.5 Hz, respectively, which may affect detection performance.

Considering the uniform distribution of eigenfrequencies, the requirements for low-frequency detection, the effectiveness of multi-band signal acquisition, as well as the compactness and aesthetic appeal of the structure, coupled structure D is identified as the optimal design.

Furthermore, by integrating the optimization strategies discussed in Section 4.1, such as shifting the supporting beam in Model A and increasing the wing size on one side of Model D, the sensor’s multi-band response capabilities can be further enhanced, leading to superior acoustic performance.

## 5. Piezoelectric Sensing Design

### 5.1. Selection of Piezoelectric Material and Operating Mode

Currently, acoustic sensors based on the inter-tympanal bridge structure primarily utilize three sensing mechanisms: optical sensing, capacitive sensing, and piezoelectric sensing. Among these, optical sensing offers the highest sensitivity; however, its complex optical system and large volume hinder miniaturization and commercial applications. Capacitive sensing, while structurally simpler, requires a bias voltage and is susceptible to residual stress during fabrication, which may cause comb finger misalignment, leading to reduced sensitivity and increased noise.

In contrast, piezoelectric sensing is easier to implement, cost-effective, and does not require a bias voltage, making it less susceptible to noise. Therefore, to achieve a compact and highly sensitive sensor, this study adopts a piezoelectric sensing approach by depositing a piezoelectric thin film on the diaphragm surface.

Common piezoelectric materials include PZT, polyvinylidene fluoride (PVDF), and AlN. Among these, PZT is the most widely used material in piezoelectric MEMS directional acoustic sensors. However, PZT exhibits high dielectric loss and acoustic loss, making it more susceptible to thermo-mechanical noise, which in turn degrades the SNR. In comparison, AlN offers lower dielectric loss and acoustic loss while being compatible with CMOS fabrication processes. This allows for effective suppression of thermo-mechanical noise, thereby improving the SNR.

Piezoelectric energy conversion is primarily achieved through two operating modes: the d₃₁ mode and the d₃₃ mode.

- In the d₃₁ mode, the direction of stress applied to the piezoelectric thin film is perpendicular to the direction in which the electric charge is generated. As a result, the electrodes must be placed on the top and bottom surfaces of the piezoelectric thin film, and the output signal amplitude is proportional to the film thickness. However, due to the thin nature of MEMS-fabricated piezoelectric thin films, the output in this mode is significantly limited.
- In contrast, the d₃₃ mode aligns the stress direction with the charge generation di-rection, requiring only IDTs on the top surface of the film. In this mode, the output signal strength can be optimized by adjusting the electrode spacing, making it independent of the film thickness.

In summary, to enhance the sensitivity and SNR of the sensor, this study employs AlN as the piezoelectric material and selects the d₃₃ mode as the operating mode to optimize the signal output performance.

### 5.2. Design of Branched Comb-Shaped Interdigitated Electrodes

**Figure 11:**
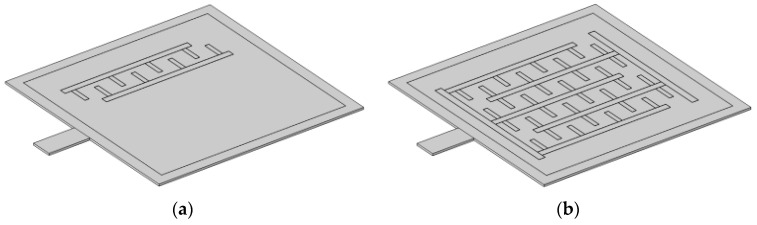
(**a**) Schematic diagram of the conventional IDE structure; (**b**) schematic diagram of the branched comb-like IDE structure.

To detect the output charge of the piezoelectric thin film in the d₃₃ operating mode, IDTs are commonly used as sensing electrodes. Traditional IDTs typically consist of a pair of vertically arranged electrodes. However, to maximize the electrode coverage on the diaphragm surface and enhance displacement-detection capabilities, this study proposes a branched comb-shaped interdigitated electrode, inspired by branched comb capacitor structures [21]. This novel design effectively increases the number of electrode pairs, thereby improving the output signal level. The schematic diagrams of conventional IDTs and branched comb-shaped IDTs are shown in Figure 11.

To validate the effectiveness of the branched comb-shaped IDTs in the d₃₃ operating mode, a multi-physics simulation was conducted using COMSOL Multiphysics. The study focused on one wing of the inner diaphragm, where a 0.5 μm thick AlN piezoelectric thin film was deposited. Additionally, a 1 μm thick aluminum (Al) electrode was placed on the AlN surface. Acoustic waves were incident from the air domain on the diaphragm surface, causing it to vibrate. The diaphragm displacement was then converted into an electrical signal via the AlN piezoelectric thin film and the branched comb-shaped IDTs. The entire process was simulated in COMSOL Multiphysics by coupling three physical fields: pressure acoustics, solid mechanics, and electrostatics.

During the simulation, accurate boundary conditions and coupling interfaces were applied across different physical domains. A frequency domain study was established to evaluate the electrical response of the branched comb-shaped IDTs under an incident sound pressure of 1 Pa at 1 kHz. A parametric sweep was performed on key structural parameters, including the width, length, and spacing of the sensing electrodes, as well as the width of the main electrodes.

By comprehensively analyzing the impact of various parameters on the performance of the branched comb-shaped interdigitated electrodes, the optimized dimensional parameters were determined as follows: the width of the sensing electrodes is 20 μm, the length of the sensing electrodes is 90 μm, the spacing between sensing electrodes is 70 μm, and the width of the main electrode is 30 μm.

**Figure 12:**
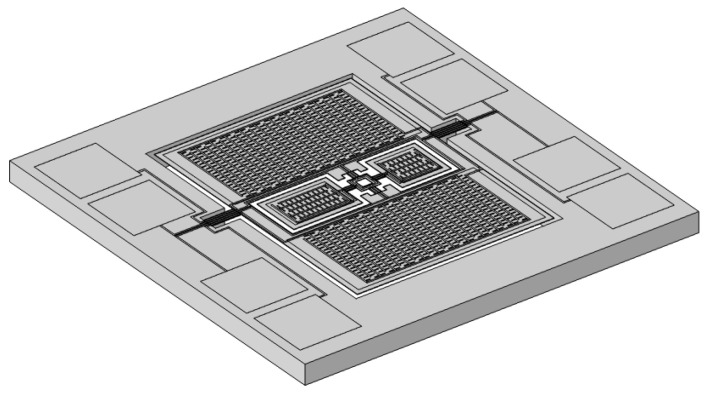
Schematic diagram of the overall sensor structure.

The final optimized sensor structure is illustrated in Figure 12.

The sensor is fabricated on a four inch silicon-on-insulator (SOI) wafer, which consists of a 400 μm thick substrate (bottom silicon), a 1 μm thick buried oxide (BOX) layer, and a 10 μm thick device layer (top silicon). A 0.2 μm thermal oxide layer is first grown via thermal oxidation. Subsequently, a 500 nm aluminum nitride (AlN) piezoelectric layer is deposited by magnetron sputtering and patterned by etching. To enhance the adhesion of the aluminum electrode layer, a 10 nm chromium (Cr) layer is sputtered before depositing the aluminum, followed by lithography and patterning to form the electrodes. The top silicon and thermal oxide layers are then etched to define the diaphragm structure. Afterward, the bottom silicon layer is etched from the backside to release the cavity and form the diaphragm. Finally, the BOX layer is removed. The materials and thickness of each layer in the sensor structure are listed in Table 3.

## 6. Simulation Validation

### 6.1. Frequency Response

**Figure 13:**
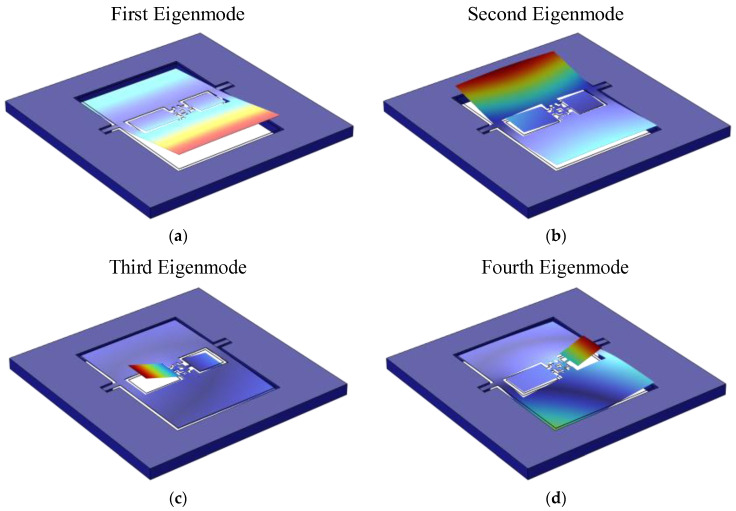
Eigenmodes of the sensor. (**a**) First eigenmode; (**b**) second eigenmode; (**c**) third eigenmode; (**d**) fourth eigenmode.

To evaluate the performance of the sensor, simulations were conducted using COMSOL Multiphysics to analyze its eigenfrequencies. The four eigenmodes are illustrated in Figure 13.

- The first eigenmode corresponds to the rocking mode of the outer diaphragm, with primary vibrations concentrated on the larger wing of the outer diaphragm.
- The second eigenmode corresponds to the bending mode of the outer diaphragm, with primary vibrations concentrated on the smaller wing of the outer diaphragm.
- The third eigenmode corresponds to the rocking mode of the inner diaphragm, with primary vibrations concentrated on the larger wing of the inner diaphragm.
- The fourth eigenmode corresponds to the bending mode of the inner diaphragm, with primary vibrations concentrated on the smaller wing of the inner diaphragm.

To ensure that all four eigenfrequencies were uniformly distributed below 2000 Hz, the influence of key structural parameters on these frequencies was further investigated. After comprehensive optimization based on the design requirements, the final sensor dimensions were determined, yielding four optimized eigenfrequencies of 405.2 Hz, 992.3 Hz, 1436.1 Hz, and 1918.6 Hz.

**Figure 14:**
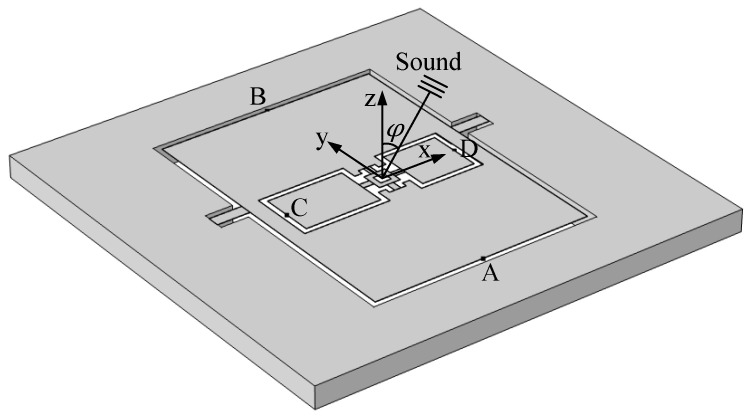
Displacement monitoring points used in frequency and directional response simulations.

Next, a frequency response simulation was conducted to evaluate the sensor’s performance under realistic operating conditions. The sensor was enclosed within a damped spherical air cavity, with an incident sound source positioned outside the cavity. Because the sensor’s dimensions were significantly smaller than the distance between the sound source and the sensor during practical testing, the far-field condition was satisfied. Therefore, in the simulation, the sound source was modeled as a plane wave with a sound pressure of 1 Pa. The angle between the sound wave and the normal of the diaphragm is denoted as *φ*. To monitor the vibrations of each wing of the internal and external diaphragms, four points A, B, C, and D were selected as displacement measurement points, as shown in Figure 14.

The definitions of points A, B, C, and D are as follows:

- Point A represents the center of the edge of the larger wing of the outer diaphragm;
- Point B represents the center of the edge of the smaller wing of the outer diaphragm;
- Point C represents the center of the edge of the larger wing of the inner diaphragm;
- Point D represents the center of the edge of the smaller wing of the inner diaphragm.

**Figure 15:**
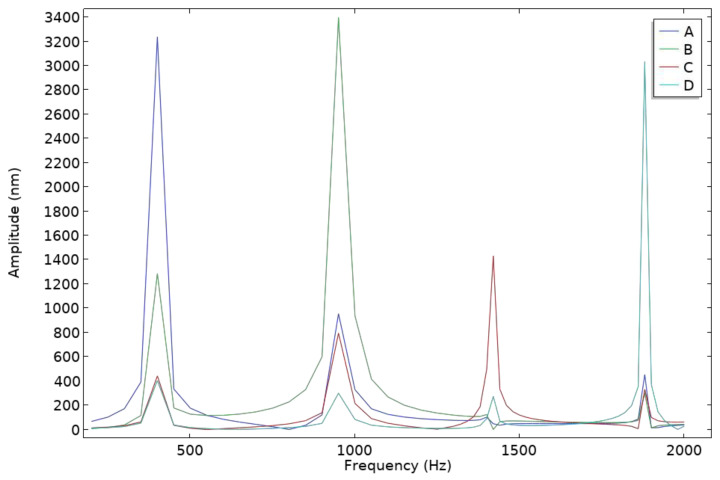
Frequency response curve of the sensor.

A fluid-structure interaction multi-physics approach was employed, coupling acoustic pressure fields with solid mechanics. The relevant boundary conditions, acoustic-structure interfaces, and material properties were accurately defined. A frequency domain study was then performed to compute the amplitude response of the sensor over the frequency range of 200–2000 Hz, and the frequency response curves were plotted, as shown in Figure 15.

As observed from the frequency response curves, the simulation results align well with expectations. Due to the influence of air damping, the four eigenfrequencies in the frequency response are slightly lower than those obtained in the eigenfrequency analysis. However, the overall trend remains consistent with the design requirements, confirming the validity of the sensor’s structural optimization.

### 6.2. Directional Response

To further evaluate the directional characteristics of the sensor, a simulation analysis of its in-plane directional response was conducted. The incident sound source was modeled as a plane wave with a sound pressure amplitude of 1 Pa. When the source angle is set to 0°, the sound wave propagates perpendicular to the sensor’s surface. The sound source was then rotated around the sensor in the x–z plane, and the sensor’s amplitude response was recorded for varying incident angles.

**Figure 16:**
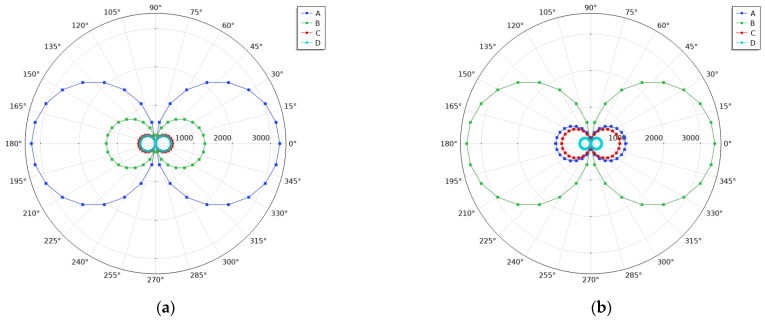

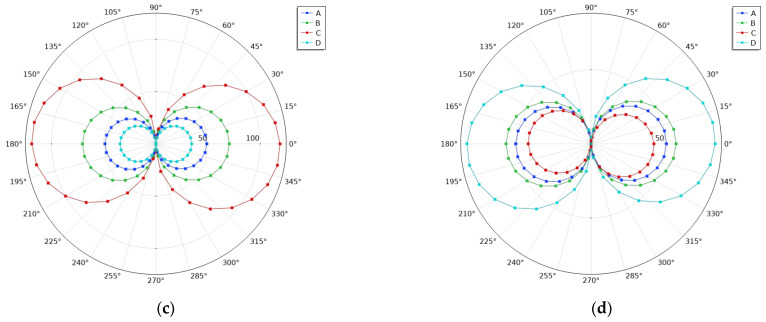
Polar plots of the sensor’s directional response at different eigenfrequencies. (**a**) First eigenfrequency; (**b**) second eigenfrequency; (**c**) third eigenfrequency; (**d**) fourth eigenfrequency.

To visually illustrate the directional characteristics at different eigenfrequencies, the computed amplitude values were represented in a polar coordinate system, and the polar response plots were generated, as shown in Figure 16.

Similarly, the definitions of points A, B, C, and D remain the same as previously described. The simulation results indicate that the amplitude response of the sensor varies with the source angle in the x–z plane, forming a uniform cosine response in the polar coordinate system. Specifically, the sensor’s amplitude reaches its maximum at 0° and 180°, while it drops to a minimum at 90° and 270°, exhibiting a directional response characteristic consistent with an ideal pressure gradient sensor.

## 7. Conclusions

This study proposes a low-frequency, multi-band piezoelectric MEMS acoustic sensor inspired by the auditory system of the *Ormia ochracea*. By introducing an improved coupling structure in the internal diaphragm, the sensor enables the detection of low-frequency signals in a compact size. The use of asymmetric wings on both the internal and external diaphragms, along with a coupled design, allows the sensor to achieve multi-band response capabilities, overcoming the limitations of conventional sensors, which typically operate in only single- or dual-frequency bands with higher eigenfrequencies. By selecting aluminum nitride with low dielectric loss and low acoustic loss as the piezoelectric material, and combining the d_33_ mode with a branched interdigital electrode structure, the sensor’s signal-to-noise ratio is enhanced. Simulation results show that the four eigenfrequencies of the sensor are evenly distributed below 2000 Hz, and at all eigenfrequencies, the sensor exhibits a uniform cosine response with respect to the incident pitch angle of the sound source.

## Figures and Tables

**Table 1:** Eigenfrequencies of each model and the reduction compared to Model A.

Structure Type	Rocking Mode Eigenfrequency (Hz)	Bending Mode Eigenfrequency (Hz)	Reduction in Rocking Mode Eigenfrequency Compared to Model A (%)	Reduction in Bending Mode Eigenfrequency Compared to Model A (%)
Model A	840.2	1902.7	0.0	0.0
Model B	832.1	1600.9	1.0	15.9
Model C	811.7	1350.3	3.4	29.0
Model D	490.8	631.4	41.6	66.8

**Table 2:** Comparison of eigenfrequencies for four coupled structures.

Coupled Structure	Eigenfrequency of Outer Diaphragm Rocking Mode (Hz)	Eigenfrequency of Outer Diaphragm Bending Mode (Hz)	Eigenfrequency of Inner Diaphragm Rocking Mode (Hz)	Eigenfrequency of Inner Diaphragm Bending Mode (Hz)
Coupled Structure A	636.0	956.7	2894.4	8458.2
Coupled Structure B	186.5	232.3	1719.7	2664.6
Coupled Structure C	189.6	246.9	2958.9	8763.4
Coupled Structure D	451.6	870.8	1728.5	2016.0

**Table 3:** Materials and thickness of each layer in the sensor.

Layer	Material	Thickness (μm)
Electrode	Al + Cr	1 + 0.01
Piezoelectric	AlN	0.5
Thermal oxide	SiO_2_	0.2
Top silicon	Si	10
BOX	SiO_2_	1
Bottom silicon	Si	400

## Data Availability

Data will be made available on request.

## Abbreviations

The following abbreviations are used in this manuscript:

MEMS	Micro-Electro-Mechanical systems
AlN	Aluminum nitride
SNR	Signal-to-noise ratio
TDOA	Time difference of arrival
ILD	Interaural level difference
ITD	Interaural time difference
PZT	Lead zirconate titanate
CMOS	Complementary metal-oxide-semiconductor
IDT	Interdigitated electrode
2-DOF	Two-degree-of-freedom
PVDF	Polyvinylidene fluoride
Al	Aluminum
SOI	Silicon-on-insulator
BOX	Buried oxide
Cr	Chromium

## References

[1] Rahaman A., Kim B. Fly-inspired mems directional acoustic sensor for sound source direction. Proceedings of the 2019 20th International Conference on Solid-State Sensors, Actuators and Microsystems & Eurosensors XXXIII (TRANSDUCERS & EUROSENSORS XXXIII).

[2] Rahaman A., Kim B., Park D. (2025). Design and characterizations of a multi–sound receiver using fly *Ormia ochracea*’s ears–inspired MEMS directional microphone array. Appl. Acoust..

[3] Zhang Y.S., Windmill J.F.C., Uttamchandani D. Biomimetic MEMS directional microphone structures for multi-band operation. Proceedings of the SENSORS, 2014 IEEE.

[4] Miles R.N., Robert D., Hoy R.R. (1995). Mechanically coupled ears for directional hearing in the parasitoid fly *Ormia ochracea*. J. Acoust. Soc. Am..

[5] Robert D., Miles R.N., Hoy R.R. (1996). Directional hearing by mechanical coupling in the parasitoid fly *Ormia ochracea*. J. Comp. Physiol. A.

[6] Ishfaque A., Kim B. (2016). Squeeze film damping analysis of biomimetic micromachined microphone for sound source localization. Sens. Actuators A Phys..

[7] Liu H.J., Yu M., Zhang X.M. (2008). Biomimetic optical directional microphone with structurally coupled diaphragms. Appl. Phys. Lett..

[8] Alves F., Rabelo R., Karunasiri G. (2022). Dual band MEMS directional acoustic sensor for near resonance operation. Sensors.

[9] Wilmott D., Alves F., Karunasiri G. (2016). Bio-inspired miniature direction finding acoustic sensor. Sci. Rep..

[10] Rabelo R.C., Alves F.D., Karunasiri G. (2020). Electronic phase shift measurement for the determination of acoustic wave DOA using single MEMS biomimetic sensor. Sci. Rep..

[11] Ishfaque A., Rahaman A., Kim B. (2019). Bioinspired low noise circular-shaped MEMS directional microphone. J. Micro/Nanolithogr. MEMS MOEMS.

[12] Rahaman A., Ishfaque A., Jung H., Kim B. (2018). Bio-inspired rectangular shaped piezoelectric MEMS directional microphone. IEEE Sens. J..

[13] Rahaman A., Kim B. (2022). An mm-sized biomimetic directional microphone array for sound source localization in three dimensions. Microsyst. Nanoeng..

[14] Zhang Y.S., Bauer R., Jackson J.C., Whitmer W.M., Windmill J.F.C., Uttamchandani D. (2018). A low-frequency dual-band operational microphone mimicking the hearing property of *Ormia ochracea*. J. Microelectromech. Syst..

[15] Miles R.N., Cui W.L., Su Q.T., Homentcovschi Q. (2014). A MEMS low-noise sound pressure gradient microphone with capacitive sensing. J. Microelectromech. Syst..

[16] Seo Y., Corona D., Hall N.A. (2017). On the theoretical maximum achievable signal-to-noise ratio (SNR) of piezoelectric microphones. Sens. Actuators A Phys..

[17] Rahaman A., Jung H., Kim B. (2021). Coupled D33 mode-based high performing bio-inspired piezoelectric MEMS directional microphone. Appl. Sci..

[18] Zhang Y.S., Bauer R., Windmill J.F.C., Uttamchandani D. Multi-band asymmetric piezoelectric MEMS microphone inspired by the *Ormia ochracea*. Proceedings of the 2016 IEEE 29th International Conference on Micro Electro Mechanical Systems (MEMS).

[19] Bauer R., Zhang Y.S., Jackson J.C., Whitmer W.M., Brimijoin W.O., Akeroyd M., Uttamchandani D., Windmill J.F.C. Housing influence on multi-band directional MEMS microphones inspired by *Ormia ochracea*. Proceedings of the 2016 IEEE SENSORS.

[20] Zhang Y.S. (2019). Development of Biomimetic MEMS Microphones Inspired by *Ormia Ochracea* Working in Audio Range. Ph.D. Thesis.

[21] Downey R.H., Karunasiri G. (2013). Reduced residual stress curvature and branched comb fingers increase sensitivity of MEMS acoustic sensor. J. Microelectromech. Syst..

